# Gut Failure During Pediatric Critical Illness: Toward a Phenotype-Driven Framework for Early Recognition, Prognostication, and Prevention

**DOI:** 10.3390/children13101433

**Published:** 2026-10-19

**Authors:** Marwa Mansour, Maria Teresa Valenzuela, Nicole Knebusch, Jorge A. Coss-Bu, Katri V. Typpo

**Affiliations:** 1 Division of Critical Care Medicine, Department of Pediatrics, Baylor College of Medicine, Houston, TX 77030, USA; 2 Texas Children’s Hospital, Houston, TX 77030, USA

**Keywords:** gastrointestinal dysfunction, gut failure, organ failure, feeding intolerance

## Abstract

Gastrointestinal dysfunction is common during critical illness, yet gut failure remains poorly defined, inconsistently diagnosed, and incompletely integrated into organ dysfunction frameworks. Current clinical approaches often recognize gut failure only after overt manifestations such as feeding intolerance, ileus, abdominal distension, gastrointestinal bleeding, or radiographic bowel injury have developed. However, these signs may represent late manifestations of distinct and potentially evolving pathophysiologic pathways. In this narrative review, we propose a phenotype-driven framework for gut failure during critical illness, with emphasis on pediatric critical care. We conceptualize gut failure as a dynamic syndrome comprising overlapping phenotypes: motility-dominant dysfunction, perfusion-ischemic injury, epithelial barrier failure, dysbiosis, absorptive-nutritional failure, treatment-modified dysfunction, and mixed evolving states. Linking these phenotypes to clinical presentation, prognosis, and management may improve early diagnosis and allow clinicians to anticipate progression before irreversible injury occurs. Pediatric patients represent a particularly vulnerable population because gut failure may affect not only short-term outcomes such as infection, mechanical ventilation, and mortality, but also nutrition delivery, growth, microbiome development, and recovery. A phenotype-based approach may shift gut failure from a late symptom-based diagnosis toward an earlier, mechanism-informed, and potentially preventable process.

## 1. Introduction

The gastrointestinal tract is increasingly recognized as a central organ in the pathobiology of critical illness [1,2,3]. Beyond digestion and absorption, the gut contributes to immune regulation, epithelial barrier function, endocrine signaling, microbial homeostasis, and communication with distant organs [4,5]. During critical illness, these functions may be disrupted by shock, hypoxemia, systemic inflammation, fluid overload, vasoactive agents, antibiotics, opioids, sedatives, parenteral nutrition, and loss of enteral substrate. The resulting syndrome is frequently described as gastrointestinal dysfunction, acute gastrointestinal injury, intestinal failure, or gut failure; however, these terms remain variably defined and inconsistently applied [3,6,7,8].

The clinical challenge is that gut failure lacks a single objective bedside measurement [1,3]. Unlike renal, respiratory, or cardiovascular dysfunction, which can be tracked using creatinine, gas exchange, or vasoactive support, gut failure is usually inferred from nonspecific signs such as vomiting, abdominal distension, high gastric residual volume, diarrhea, constipation, feeding intolerance, gastrointestinal bleeding, or intra-abdominal hypertension [5,9,10,11].

The available gastrointestinal dysfunction scoring tools vary substantially, often lack objective biomarkers, and remain insufficiently validated in critical illness [12]. This diagnostic ambiguity has important consequences. Current practice often identifies gut failure only after overt dysfunction has occurred. Yet, clinical manifestations such as feeding intolerance or ileus may represent the end result of different biologic processes [13,14,15]. A patient with medication-associated ileus, a patient with shock-associated splanchnic hypoperfusion, and a patient with antibiotic-driven dysbiosis may all be labeled as having feeding intolerance, but their prognosis and management differ substantially. Therefore, the main premise of this review is that improved understanding of gut failure phenotypes may allow clinicians to predict risk, diagnose earlier, and implement targeted interventions to prevent or attenuate progression during critical illness.

This manuscript aims to propose a conceptual framework where gut failure should be approached not only as a severity-based syndrome but also as a phenotype-based process to help with clinical identification and management. Severity scores such as acute gastrointestinal injury (AGI) and gastrointestinal dysfunction score (GIDS) may help identify the burden of dysfunction [16,17], whereas phenotyping may help explain why the gut is failing and what can be modified. This framework is especially relevant in pediatric critical care, where gut dysfunction may influence nutrition delivery, growth, immune function, microbiome development, and long-term recovery. Pediatric-specific evidence was prioritized throughout this review. However, because validated pediatric data on gut failure definitions, phenotypes, diagnostic tools, and management strategies remain limited, relevant adult critical care literature was also included. Adult studies were used primarily to inform general concepts of gastrointestinal dysfunction, scoring systems, pathophysiology, and bedside assessment, while pediatric studies were emphasized when addressing pediatric-specific outcomes, nutrition delivery, microbiome development, sepsis, ECMO, and fluid overload.

## 2. Methods

This narrative review and conceptual framework were developed through a structured literature search focused on gastrointestinal dysfunction and gut failure during critical illness conducted between January 2005 and June 2026. We searched PubMed, Embase, the Cochrane Library, and OpenEvidence using combinations of key terms including “gut failure,” “gastrointestinal dysfunction,” “acute gastrointestinal injury,” “feeding intolerance,” “intestinal failure,” “critical illness,” “pediatric critical care,” “enteral nutrition,” “dysbiosis,” “microbiome,” and “short-chain fatty acids.” The search prioritized studies addressing definitions and scoring systems, pathophysiology, bedside diagnosis, clinical outcomes, nutrition delivery, microbiome disruption, and management strategies relevant to critically ill patients. We included adult critical care literature when pediatric-specific evidence was limited, particularly for definitions, scoring systems, and mechanistic concepts. Pediatric studies were prioritized when discussing nutrition delivery, microbiome development, sepsis, ECMO, fluid overload, and pediatric-specific outcomes. Relevant review articles, observational studies, clinical trials, systematic reviews, and expert consensus statements were reviewed. The findings were synthesized narratively to develop a conceptual phenotype-driven framework linking dominant mechanisms of gut failure to clinical presentation, prognosis, and management priorities. Articles were excluded if they were not relevant to critical illness, focused exclusively on chronic intestinal failure outside the ICU setting, were not available in English, or did not address gastrointestinal dysfunction, gut failure, nutrition delivery, or related mechanisms. Because this was not designed as a systematic review, a formal count of screened and included records was not performed. This phenotype-driven framework is proposed as a conceptual and clinically informed model. It has not yet been prospectively validated and reflects synthesis of the available literature combined with the authors’ clinical experience in caring for critically ill children.

## 3. Evolution of Definitions and Scoring Systems

Historically, gut failure has been difficult to define because the gut performs several integrated functions rather than one easily measurable task. Intestinal failure has traditionally been defined as the inability to maintain adequate nutrition, fluid, or electrolyte balance due to anatomical or physiological gastrointestinal dysfunction. Grainger et al. described intestinal failure as the inability to maintain nutrition or fluid and electrolyte balance due to gastrointestinal disease, with classifications based on duration and reversibility [13,18].

In the ICU, the term “gut failure” is broader and often refers to acute disruption of motility, barrier function, perfusion, absorption, immune signaling, and microbial ecology. The PODIUM (Pediatric Organ Dysfunction Information Update Mandate) consensus criterion for severe gastrointestinal dysfunction in critically ill children reflects signs and symptoms of GI dysfunction due to GI ischemia, defined as the presence of any one of the following: Bowel perforation, pneumatosis intestinalis, bowel ischemia, or rectal sloughing of gut mucosa. Although it is a valuable input, it remains a weak recommendation based on expert consensus, because the supporting pediatric literature is limited [1].

The European Society of Critical Care Medicine (ESICM) Working Group introduced the AGI grading system to standardize recognition of gastrointestinal dysfunction in critically ill patients. This represented a major conceptual advance because it acknowledged gastrointestinal dysfunction as an organ injury syndrome. However, AGI remains primarily clinical and severity-based. GIDS was later developed to align gastrointestinal dysfunction more closely with organ failure scoring approaches, yet the field still lacks consensus on the optimal assessment tool [5,9,15].

In a prospective observational study, Asrani et al. found that gastrointestinal dysfunction was highly prevalent among critically ill adults: all enrolled patients had at least one gastrointestinal sign or symptom, severe dysfunction occurred in a substantial proportion, and severe dysfunction was associated with longer ICU stay and mechanical ventilation duration. Elevated lactate, positive fluid balance, gut-related admission diagnosis, aggressive enteral feeding, and multiple inotropes were associated with greater gastrointestinal dysfunction severity. These findings support the importance of systematic assessment but also highlight the need for more objective and mechanism-specific tools [12,19].

The limitations of current scoring systems are particularly relevant to pediatrics. Most AGI and GIDS data derive from adult populations, and pediatric validation remains limited. Children differ from adults in intestinal maturation, microbiome composition, immune development, nutritional reserves, growth requirements, and hemodynamic physiology.

Thus, pediatric gut failure requires a framework that incorporates not only mortality and ICU length of stay but also nutrition adequacy, growth, microbial development, and recovery [3,12,20].

## 4. Pathophysiology of Gut Failure During Critical Illness

Gut failure during critical illness is not a single disease process. It reflects the convergence of multiple, often overlapping mechanisms. These include splanchnic hypoperfusion, microcirculatory dysfunction, epithelial injury, altered permeability, immune dysregulation, dysmotility, dysbiosis, impaired absorption, and iatrogenic exposures [6,7,21].

The gut is particularly vulnerable during shock because splanchnic blood flow may be reduced to preserve perfusion of vital organs. Hypoperfusion, vasoconstriction, venous congestion, and reperfusion injury can impair mucosal oxygenation and epithelial integrity. Fluid overload may further worsen gut function by causing splanchnic congestion, bowel wall edema, impaired gastrointestinal perfusion and motility, and increased risk of bowel ischemia or abdominal compartment syndrome [22,23,24,25].

Critical illness also disrupts epithelial barrier function. Under normal conditions, the intestinal epithelium separates the host from luminal microbes while allowing regulated absorption and immune signaling. During critical illness, epithelial apoptosis, tight junction disruption, mucus layer injury, and impaired mucosal immunity may increase permeability. This process provides biologic plausibility for the long-standing concept of the gut as a contributor to systemic inflammation and multiple organ dysfunction [8,13].

The microbiome is another key component of gut function. Dysbiosis in critical illness is a potential form of organ failure, characterized by loss of health-promoting commensals and expansion of potentially pathogenic taxa. Dysbiosis may alter gut integrity, short-chain fatty acid production, bile acid metabolism, and gut–organ signaling [26,27,28].

Pediatric studies support the relevance of microbiome disruption. Evidence shows that critically ill children had reduced gastrointestinal and tongue microbial diversity, enrichment of pathogens such as Enterococcus and Staphylococcus, and depletion of commensals such as Faecalibacterium and Ruminococcus [29,30,31]. In pediatric sepsis, Weiss et al. demonstrated reduced microbial diversity and altered short-chain fatty acid profiles, including decreased butyrate-related metabolites, suggesting a potential link between dysbiosis and immunometabolic dysfunction [32]. Sankar et al. similarly reported reduced alpha diversity, altered Firmicutes/Bacteroidetes ratios, clinical gastrointestinal dysfunction, and reduced detectable stool butyrate among children with sepsis or septic shock [33].

## 5. A Phenotype-Driven Framework of Gut Failure

**Figure 1:**
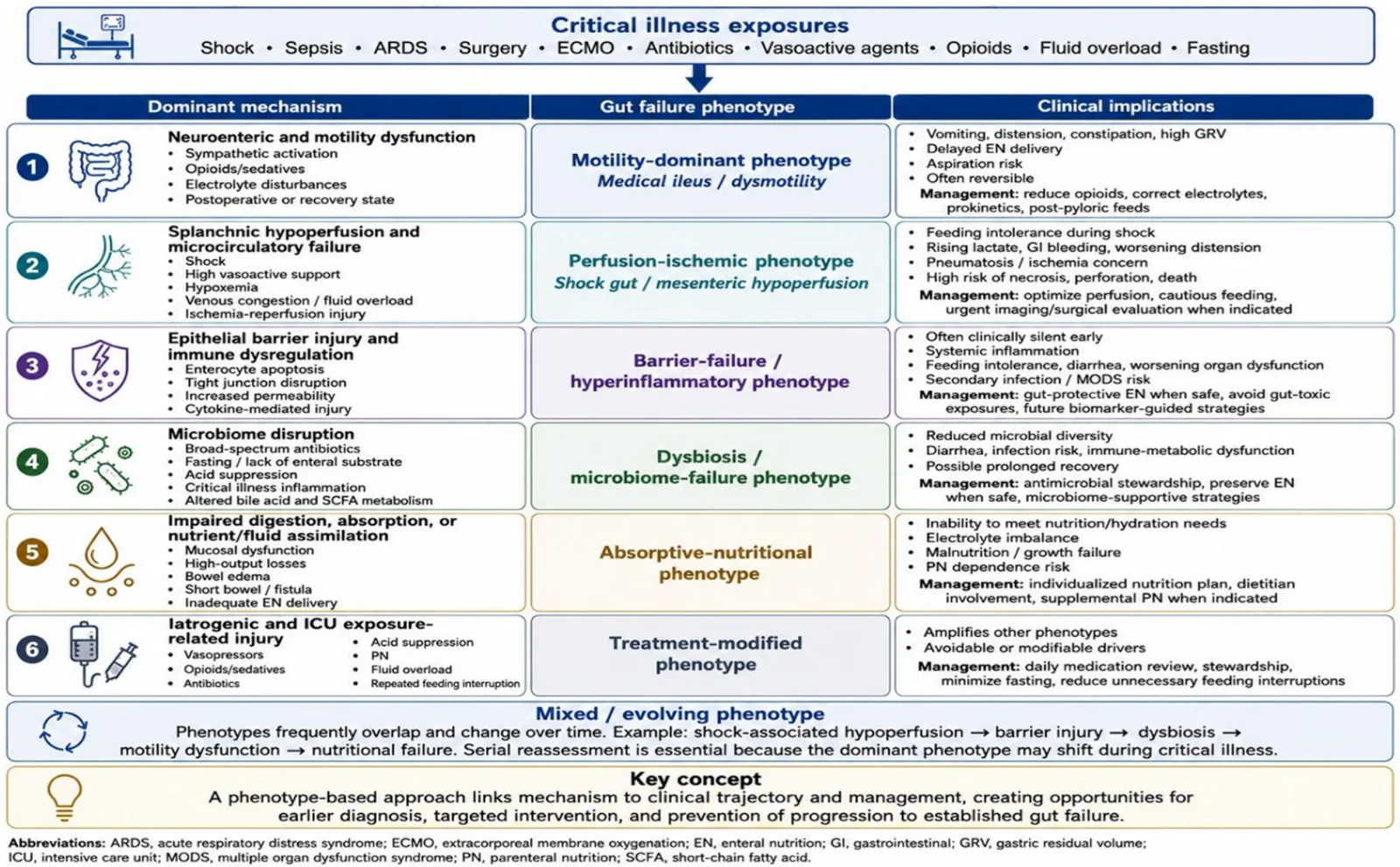
Phenotype-based framework of gut failure during critical illness.

We propose a conceptual, phenotype-driven approach that defines gut failure by dominant mechanism, clinical pattern, trajectory, and therapeutic implications. This framework has not been clinically validated and does not replace the existing gut failure scores. These phenotypes are not mutually exclusive. A patient may transition from one phenotype to another over time or may express several simultaneously (Figure 1).

### 5.1. Motility-Dominant Phenotype: Medical Ileus and Neuroenteric Dysmotility

The motility-dominant phenotype is one of the most clinically visible forms of gut dysfunction in the ICU. It is characterized by impaired coordination of gastric, small bowel, or colonic motility, resulting in delayed gastric emptying, high gastric residual volumes, vomiting, abdominal distension, constipation, ileus, or diarrhea. In critically ill children, gastrointestinal motility depends on a complex interaction among the autonomic nervous system, enteric nervous system, interstitial cells of Cajal, and enteroendocrine signaling. Critical illness disrupts this coordination through sympathetic activation, parasympathetic suppression, inflammation, sedatives, opioids, neuromuscular blockade, electrolyte abnormalities, and nonphysiologic feeding patterns.

This phenotype is frequently encountered in mechanically ventilated children, postoperative patients, patients receiving high-dose opioids or sedatives, neuromuscular blockades, electrolyte imbalances, and those recovering from shock or systemic inflammation [3,34].

Although it may delay achievement of enteral nutrition targets and increase the risk of aspiration or feeding interruptions, its trajectory is often reversible when the underlying illness improves, and modifiable contributors are addressed. Management is therefore directed toward restoration of coordinated motility: minimizing opioids when feasible, correcting electrolyte abnormalities, implementing bowel regimens, considering prokinetic therapy, and using post-pyloric feeding in patients with persistent gastric intolerance. Nutrition protocols may also reduce practice variability and improve time to feeding initiation and goal achievement in critically ill children, although pediatric outcome data remain limited.

The prognostic implication of this phenotype is usually related less to direct mortality and more to nutritional debt, prolonged ventilation, aspiration risk, and ICU length of stay. Importantly, the motility-dominant phenotype should be distinguished from ischemic or inflammatory phenotypes because management priorities differ. Escalating feeds in a child with isolated medication-related ileus may be appropriate after mitigation of reversible factors, whereas feeding intolerance during uncontrolled shock may represent an early warning sign of hypoperfusion [3,35,36].

### 5.2. Perfusion-Ischemic Phenotype: Shock Gut and Mesenteric Hypoperfusion

The perfusion-ischemic phenotype reflects splanchnic hypoperfusion, microcirculatory dysfunction, vasoconstriction, venous congestion, or ischemia–reperfusion injury. It is most relevant in septic shock, cardiogenic shock, severe hypoxemia, high vasoactive support, postcardiac surgery, and extracorporeal membrane oxygenation (ECMO) [33,37].

Clinically, this phenotype should be suspected when feeding intolerance occurs with escalating vasoactive support, rising lactate, worsening acidosis, abdominal distension, gastrointestinal bleeding, pneumatosis, portal venous gas, or signs of abdominal compartment syndrome. It carries the highest immediate risk because it may progress to bowel necrosis, perforation, sepsis, and death [21,38,39].

Pediatric ECMO illustrates the tension between gut protection and feeding risk. Alexander et al. found that initiation of enteral nutrition by day 3 of ECMO and lower vasoactive-inotropic score at feeding initiation were associated with survival, while also acknowledging that vasoactive support and hemodynamic instability often delay feeding because of concern for reduced splanchnic blood flow [40]. A systematic review found that enteral nutrition in children receiving ECMO did not appear to increase mortality or gastrointestinal complications compared with parenteral nutrition. However, available studies were heterogeneous and largely observational [41].

### 5.3. Barrier-Failure and Hyperinflammatory Phenotype

The barrier-failure phenotype is characterized by disruption of the intestinal epithelial interface that normally separates the host from luminal microbes and microbial products. Critical illness increases epithelial apoptosis, suppresses crypt proliferation, alters tight junction integrity, and increases intestinal permeability. The result is a gut barrier that is less capable of maintaining selective absorption and microbial containment. This phenotype provides the biologic foundation for the long-standing concept of the gut as a driver of systemic inflammation and multiorgan dysfunction syndrome (MODS) [21,39].

Unlike motility dysfunction, barrier failure may be clinically silent early in its course. Patients may not initially have overt abdominal findings, yet may develop systemic inflammation, immune dysregulation, secondary infections, or worsening organ dysfunction. Over time, clinical manifestations may include feeding intolerance, diarrhea, abdominal distension, worsening sepsis physiology, or persistent inflammatory critical illness. Biomarkers such as intestinal fatty acid binding protein, citrulline, D-lactate, tight junction proteins, and microbial products have been proposed as markers of enterocyte injury, epithelial mass, permeability, or bacterial translocation, but none are currently established for routine bedside classification of this phenotype [12,19].

Management remains largely supportive and prevention-focused. Early enteral nutrition, when hemodynamically safe, may help preserve epithelial integrity, stimulate motility, support mucosal immune function, and provide luminal substrate for the microbiome. In pediatric ARDS, nutrition has been proposed as a primary therapy rather than merely supportive care because of its potential effects on epithelial barrier function, intestinal dysbiosis, lean body mass preservation, and systemic inflammation. Future management of this phenotype will likely depend on biomarker-guided risk stratification and targeted therapies aimed at preserving epithelial integrity, reducing permeability, and modulating maladaptive inflammation.

### 5.4. Dysbiosis or Microbiome-Failure Phenotype

Clinically, the dysbiosis phenotype may present with diarrhea, feeding intolerance, recurrent or nosocomial infections, persistent inflammation, or poor recovery. However, like barrier failure, it may be difficult to recognize without microbial or metabolic profiling. Management strategies include antibiotic stewardship, minimizing unnecessary acid suppression, avoiding prolonged fasting when enteral nutrition is safe, and considering microbiome-supportive nutrition strategies. Current evidence does not yet support routine microbiome-directed therapy for all critically ill children, but this phenotype is likely to become increasingly important as metabolomic and microbiome-based diagnostics become more feasible [32,42].

The dysbiosis phenotype reflects disruption of the intestinal microbial ecosystem and its metabolic functions. In health, the gut microbiome contributes to colonization resistance, immune development, epithelial homeostasis, bile acid metabolism, and production of short-chain fatty acids (SCFAs). During critical illness, this ecosystem may rapidly lose diversity, become depleted of commensal obligate anaerobes, and shift toward dominance by potentially pathogenic organisms. Contributing factors include broad-spectrum antibiotics, acid suppression, opioids, fasting, parenteral nutrition, hypoperfusion, inflammation, altered oxygen tension, and prolonged ICU exposure [32,43,44].

Pediatric data increasingly support the relevance of this phenotype. Children with sepsis and septic shock have been shown to have reduced microbial diversity compared with healthy controls, depletion of beneficial taxa such as Bifidobacterium, dominance by organisms such as Bacteroides, Enterobacteriaceae, and Enterococcaceae, and reduced SCFA concentrations. Lower Firmicutes/Bacteroidetes ratios have also been associated with clinical GI dysfunction, inflammatory markers, and higher organ failure scores. In a separate pediatric sepsis cohort, intestinal dysbiosis was associated with altered SCFA profiles, including decreased plasma butyrate, which was further associated with impaired mitochondrial respiration in peripheral blood mononuclear cells and altered immune function markers [32,33,45].

The dysbiosis phenotype is important because it may be modifiable. Enteral substrate, fiber, prebiotics, probiotics, antibiotic stewardship, and avoidance of unnecessary acid suppression may eventually become phenotype-specific therapies. However, current evidence does not support indiscriminate microbiome-directed treatment in all critically ill children [42,46,47].

### 5.5. Absorptive-Nutritional Failure Phenotype

This phenotype is a sequela of a multifactorial process leading to failure of the gut to maintain hydration, electrolyte balance, macronutrient delivery, micronutrient sufficiency, or growth. This phenotype may occur transiently during critical illness due to mucosal injury, dysmotility, diarrhea, high-output stomas or fistulas, pancreaticobiliary dysfunction, bowel edema, or prolonged inability to deliver enteral nutrition. In a prospective comparative study, Duodenal biopsies in enterally fed ICU patients show mild-to-moderate epithelial injury [48]. Inflammation and oxidative stress reduce tight-junction protein expression (downregulated ZO-1, upregulated claudin-2, MLCK activation), accelerate enterocyte apoptosis, and cause mucosal atrophy, collectively increasing permeability and reducing effective absorptive surface [39,48,49].

### 5.6. Iatrogenic and Exposure-Driven Gut Failure

This is not a distinct phenotype. ICU therapies may alter gut physiology and amplify underlying injury. Vasopressors may worsen splanchnic hypoperfusion in vulnerable patients; opioids and sedatives impair motility; antibiotics and acid suppression disrupt microbial ecology; prolonged fasting reduces luminal substrate; and parenteral nutrition may alter microbial colonization patterns. In infants receiving total parenteral nutrition, longitudinal microbiome studies show reduced bacterial diversity and altered colonization patterns over time, including lower Bacteroides and Bifidobacterium abundance [37].

Antibiotic exposure is particularly important because it can produce both short-term dysbiosis and longer-term expansion of antimicrobial resistance genes within the gut microbiome. Studies in heavily antibiotic-exposed populations demonstrate that broad-spectrum antibiotics are associated with marked microbial disruption and increased resistome features. This has important implications for critically ill children, who often receive broad-spectrum antibiotics early and repeatedly. The treatment-modified phenotype, therefore, emphasizes that management choices are not neutral; they may either preserve or further disrupt gut function.

Opioids and sedatives impair motility. Antibiotics and acid suppression disrupt microbial ecology. Parenteral nutrition and fasting reduce enteral substrate [50,51].

Management of this phenotype is based on harm reduction: antimicrobial stewardship, daily reassessment of acid suppression, opioid-sparing analgesia when feasible, avoidance of unnecessary fasting, early enteral substrate when safe, careful fluid balance, and thoughtful use of parenteral nutrition. This phenotype may overlap with all others and is best understood as a modifier of gut failure trajectory rather than a stand-alone diagnosis.

Fluid overload worsens bowel edema and perfusion. These exposures can amplify existing phenotypes [52,53].

### 5.7. Proposed Conceptual Model

In clinical practice, gut failure rarely follows a single pathway. The proposed phenotype framework is intended to recognize the major mechanisms of gut failure while also acknowledging that these mechanisms often overlap and evolve over time. A critically ill child may initially develop perfusion-related injury, then progress to epithelial barrier dysfunction, dysbiosis related to antibiotics, opioid-associated ileus, and nutritional deficits from repeated feeding interruptions. Therefore, the dominant phenotype may change during the ICU course, and management should be reassessed accordingly.

This dynamic nature highlights the limitations of static severity scores. Existing scoring systems can describe the severity of gastrointestinal dysfunction at a specific time point, but they do not necessarily identify the underlying mechanism or explain whether the process is improving, worsening, or transitioning. A phenotype-based approach encourages serial reassessment of the clinical context, including perfusion, motility, barrier injury, dysbiosis, absorptive capacity, and treatment-related contributors. This may help clinicians align management with the dominant mechanism rather than relying on nonspecific labels such as feeding intolerance or gut dysfunction.

Taken together, these phenotypes support a multidimensional model of gut failure during critical illness. Some phenotypes, such as motility-dominant dysfunction, may be more reversible and primarily limit nutrition delivery. Others, such as perfusion-ischemic injury, may carry higher risk and require urgent recognition. Barrier failure, dysbiosis, and absorptive-nutritional failure may contribute to systemic inflammation, infection risk, nutritional deficits, and delayed recovery. ICU-related therapies and exposures should be considered important modifiers that may precipitate, worsen, or mimic these phenotypes rather than representing a distinct phenotype by themselves.

## 6. Clinical Diagnosis and Early Recognition

**Figure 2:**
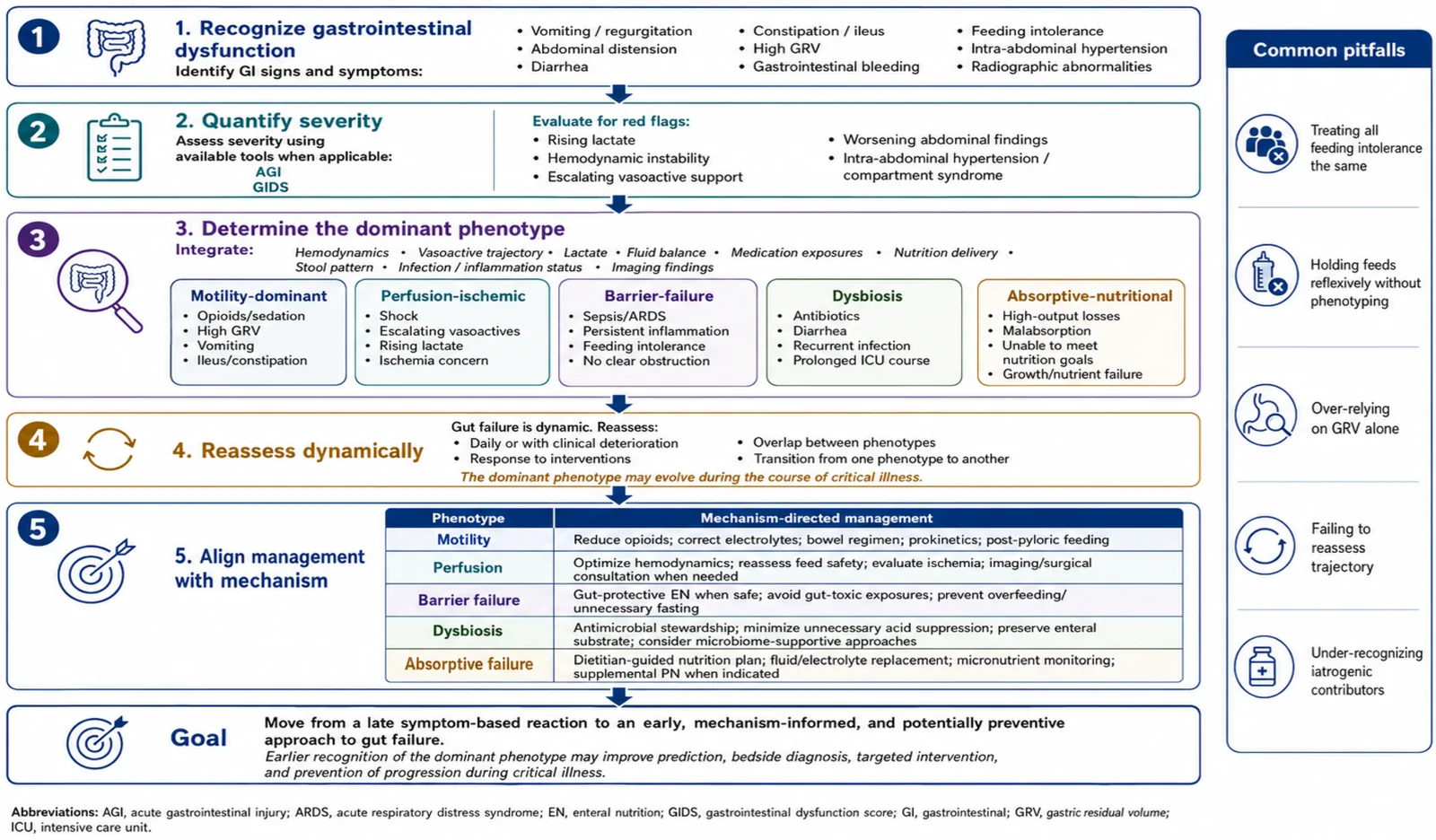
Five-step bedside phenotype-based approach to gut failure during critical illness.

The central clinical goal is to move gut failure from a late diagnosis to an earlier, mechanism-informed process. Early recognition requires a structured bedside assessment that combines symptoms, severity scoring, trajectory, and phenotype interpretation (Figure 2).

We propose a practical approach that includes five steps. First, identify gastrointestinal signs: vomiting, distension, diarrhea, constipation, high gastric residuals, bleeding, feeding intolerance, intra-abdominal hypertension, or radiographic abnormalities. Second, grade severity using available tools such as AGI or GIDS when applicable. Third, identify the dominant phenotype by integrating hemodynamics, vasoactive trajectory, lactate, fluid balance, medication exposures, nutrition delivery, stool pattern, infection status, and imaging. Fourth, reassess dynamically because gut failure evolves over time. Fifth, align management with mechanism rather than responding reflexively by holding feeds.

This distinction matters. Feeding intolerance during opioid exposure in a hemodynamically improving child suggests a motility phenotype. Feeding intolerance during escalating shock suggests a perfusion-ischemic phenotype. Diarrhea after broad-spectrum antibiotics may suggest dysbiosis. Persistent inability to meet nutrition goals despite hemodynamic recovery may suggest absorptive-nutritional failure or process-related feeding barriers.

The better we understand these phenotypes, the earlier we may be able to predict who is at risk, diagnose the dominant mechanism, and implement targeted strategies to prevent or attenuate gut failure.

## 7. Prognosis and Clinical Outcomes

Gut failure is both a marker and a potential mediator of adverse outcomes. Severe gastrointestinal dysfunction was associated with longer ICU stay and longer duration of mechanical ventilation [19,43]. Elevated lactate, positive fluid balance, multiple inotropes, and aggressive enteral feeding were associated with more severe dysfunction. These findings support the idea that gut dysfunction reflects systemic severity but may also be influenced by modifiable factors.

Pediatric outcomes must be viewed through a broader lens than mortality alone. Gut failure may contribute to nutrition debt, infections, ventilator dependence, delayed rehabilitation, altered growth, microbiome disruption, and long-term recovery challenges [54,55]. Fluid accumulation is one important modifier. Lintz et al. found that fluid accumulation in critically ill children was associated with increased mortality, acute kidney injury, prolonged mechanical ventilation, and longer ICU stay [56]. It was further described that gastrointestinal consequences of fluid overload include splanchnic congestion, intestinal edema, impaired motility, gastrointestinal bleeding, bowel ischemia, and abdominal compartment syndrome [22].

Microbiome research showed that critically ill children exhibit loss of microbial diversity, loss of site specificity, pathogen dominance, and depletion of beneficial commensals. While causality remains uncertain, these findings support future studies evaluating whether dysbiosis predicts infection, immune dysfunction, persistent inflammation, or recovery trajectory [27,30,57,58].

## 8. Management and Prevention

We suggest phenotype-directed management (Table 1).

For motility-dominant dysfunction, the priority is to restore gastrointestinal transit and avoid unnecessary nutrition interruption. This includes correction of electrolytes, reduction in opioids, bowel regimens, prokinetics when appropriate, and post-pyloric feeding in persistent gastric intolerance.

For perfusion-ischemic dysfunction, the priority is gut perfusion. Enteral nutrition should be delayed, reduced, or held in uncontrolled shock, escalating vasoactive support, rising lactate, worsening acidosis, abdominal compartment physiology, gastrointestinal bleeding, or suspected ischemia. Conversely, stable patients receiving low or moderate vasoactive support may tolerate cautious low-dose enteral nutrition. Preiser et al. suggested that low-dose enteral nutrition can be started within 48 h in selected critically ill patients, including some receiving small or moderate vasopressor doses, while warning that abdominal pain, distension, gastrointestinal paralysis, or rising abdominal pressure may indicate lower gastrointestinal intolerance [34].

For barrier-failure physiology, management is preventive: preserve perfusion, avoid unnecessary fasting, provide enteral substrate when safe, prevent overfeeding, and limit gut-toxic exposures. In experimental models, enteral feeding benefited the gut mucosal barrier during sepsis, and fasting was associated with increased gut permeability [25,29].

For dysbiosis, management should emphasize antimicrobial stewardship, minimizing unnecessary acid suppression, preserving enteral nutrition when safe, and cautious exploration of microbiome-directed therapies [43,59]. Probiotics are promising but not ready for routine universal use. The use of probiotics has been associated with reduction in ventilator-associated pneumonia (VAP) and diarrhea among mechanically ventilated children [60,61]. However, pediatric reviews caution that safety and efficacy remain population- and strain-specific [42,47,62].

For absorptive-nutritional failure, the priority is to prevent nutritional debt while avoiding overfeeding. Dietitian-led protocols, early nutrition risk assessment, protein adequacy, fluid and electrolyte monitoring, micronutrient surveillance, and individualized use of parenteral nutrition are essential [63,64,65].

## 9. Pediatric Considerations

Children are uniquely vulnerable to gut failure because of developmental differences in intestinal physiology, immune maturation, microbial ecology, and nutritional reserve. The infant and young child microbiome is dynamic, and early-life disruptions from antibiotics, illness, diet, and hospitalization may alter microbial development [65,66,67]. Previous studies have emphasized that the period from birth to 3 years is a sensitive window for microbiome and gut–brain axis development [68]. This does not imply that PICU dysbiosis inevitably causes long-term neurodevelopmental harm, but it highlights the need to evaluate outcomes beyond discharge.

Pediatric surgical patients also challenge traditional assumptions about gut rest. A meta-analysis found that early enteral nutrition within 48 h after bowel anastomosis surgery did not increase the risk of anastomotic leak, abdominal distension, wound dehiscence, or vomiting, and was associated with fewer surgical site infections, septic complications, and overall complications [69]. These data support the concept that early gut stimulation may be safe and beneficial in selected children.

In pediatric ECMO, feeding decisions require careful integration of perfusion risk, vasoactive trajectory, bleeding risk, and nutrition goals. The available evidence suggests that enteral nutrition can be feasible in selected patients and may be associated with improved outcomes, but standardized definitions of “clinical stability” and feeding safety thresholds remain needed [41].

## 10. Future Directions

Future research should focus on transforming gut failure from a late clinical diagnosis into an early, measurable, and modifiable syndrome. First, pediatric-specific definitions and scoring systems are needed. These should incorporate age-specific feeding tolerance, stool output, intra-abdominal pressure, fluid balance, biomarkers, nutrition adequacy, and outcomes.

Second, biomarker research may be phenotype-specific. Citrulline may reflect enterocyte mass, intestinal fatty acid-binding protein may reflect epithelial injury, lactate may support concern for hypoperfusion, and short-chain fatty acids or bile acids may reflect microbial metabolic function. A multimarker panel may ultimately be more useful than a single test [70,71,72,73].

Third, microbiome and metabolomic profiling should be linked to clinical decisions. Studies should determine whether dysbiosis predicts infection, immune dysfunction, organ failure, nutrition intolerance, or long-term outcomes, and whether interventions can modify these trajectories [74,75,76].

Fourth, clinical trials may be phenotype-enriched. Prokinetic trials may target motility-dominant dysfunction. Feeding during vasoactive support may be studied in perfusion-stratified groups. Microbiome interventions may be tested in children with demonstrable dysbiosis or metabolomic depletion. Nutrition trials should account for illness phase, route, dose, protein delivery, and fluid balance.

Finally, long-term pediatric outcomes should be incorporated. Growth, nutritional recovery, functional status, microbiome restoration, neurodevelopment, and post-intensive care morbidity are essential endpoints for children.

## 11. Conclusions

Current severity-based tools have improved recognition but do not fully explain the mechanism, trajectory, or therapeutic priorities.

We propose a conceptual phenotype-driven framework that may move gut failure from a late, symptom-based diagnosis toward earlier prediction, mechanism-informed diagnosis, and targeted prevention. Prospective validation in critically ill children is needed. The body of literature is lacking in pediatrics, which is a major limitation for early diagnosis and treatment.

## Figures and Tables

**Table 1:** Phenotype-driven framework of gut failure during critical illness.

Phenotype	Dominant Mechanism	Clinical Picture	Common Setting	Prognostic Implication	Management Focus
Motility-dominant	Neuroenteric and enteroendocrine dysregulation	Ileus, vomiting, high GRV, distension, constipation, diarrhea	Opioids, sedation, mechanical ventilation, postoperative state	Delayed EN, aspiration risk, longer ICU course; often reversible	Reduce opioids, correct electrolytes, prokinetics, bowel regimen, post-pyloric feeds
Perfusion-ischemic	Splanchnic hypoperfusion, microcirculatory failure, ischemia–reperfusion	Rising lactate, distension, GI bleeding, pneumatosis, bowel necrosis	Shock, high vasoactives, ECMO, severe hypoxemia	High risk of MODS, perforation, surgery, death	Optimize perfusion, cautious feeding, imaging, surgical consultation
Barrier-failure/hyperinflammatory	Enterocyte apoptosis, tight junction injury, permeability	Often silent early; systemic inflammation, infection risk, feeding intolerance	Sepsis, ARDS, burns, trauma	MODS, persistent inflammation, secondary infection	Early EN when safe, avoid gut-toxic exposures, future biomarker-guided therapies
Dysbiosis/microbiome failure	Loss of diversity, pathobiont expansion, reduced SCFAs	Diarrhea, infection risk, immune dysfunction, poor recovery	Antibiotics, acid suppression, fasting, prolonged ICU stay	Nosocomial infection, immune-metabolic dysfunction, prolonged illness	Stewardship, enteral substrate, minimize unnecessary PPIs, future prebiotic/synbiotic approaches
Absorptive-nutritional	Impaired nutrient/fluid absorption or inadequate EN delivery	Malnutrition, electrolyte loss, growth failure, high-output losses	ECMO, SBS, fistula, mucosal injury, prolonged critical illness	Nutritional debt, PN dependence, delayed recovery	Dietitian-led plan, EN optimization, PN when needed, micronutrient monitoring
Treatment-modified	ICU exposures worsening gut physiology	Variable: ileus, dysbiosis, feeding intolerance, infection	Antibiotics, opioids, sedatives, PPIs, PN, fasting	May amplify other phenotypes	Harm reduction, daily medication review, avoid unnecessary fasting, stewardship

This framework does not replace AGI, GIDS, or other severity scores; rather, it complements them. Severity scores identify the burden of gastrointestinal dysfunction, while phenotypes may elucidate the underlying biology and therapeutic priorities. Future studies should validate whether these phenotypes are reproducible, whether they predict outcomes beyond existing scores, and whether phenotype-targeted interventions can improve clinically meaningful outcomes in critically ill children.

## Data Availability

No new data were created or analyzed in this study. Data sharing is not applicable to this article.

## Abbreviations

The following abbreviations are used in this manuscript:

AGI	acute gastrointestinal injury
GIDS	gastrointestinal dysfunction score
ESICM	European Society of Critical Care Medicine
ECMO	extracorporeal membrane oxygenation
MODS	multiorgan dysfunction syndrome
SCFAs	short-chain fatty acids
VAP	ventilator-associated pneumonia
